# The Emerging Role of α-Synuclein Strains in Parkinson’s Disease: From Molecular Pathology to Clinical Phenotypes

**DOI:** 10.7759/cureus.117292

**Published:** 2026-10-01

**Authors:** Akash Verma, P C Tiwari, Brijesh Sharma, Bipin Bihari, Rishi Pal

**Affiliations:** 1 Institute of Biomedical Education and Research, Department of Pharmacy, Mangalayatan University, Aligarh, IND; 2 Department of Pharmacology and Therapeutics, King George's Medical University, Lucknow, IND; 3 Department of Pharmaceutical Chemistry, Seth Vishambhar Nath Institute of Pharmaceutical Sciences, Lucknow, IND

**Keywords:** parkinson’s disease, protein misfolding, retrospective study, snca strains, synucleinopathies

## Abstract

Progressive loss of dopaminergic neurons and the formation of α-synuclein aggregates are hallmarks of Parkinson’s disease. Biochemical, biophysical, cellular, animal, and clinical studies provide increasing evidence that α-synuclein is a highly “heterogeneous protein” that may exist in nature in different conformational states or “strains.” The various strains differ biochemically and biophysically, elicit different seeding activities in cell culture assays, and have distinct pathological and cellular effects. The ability of different α-synuclein strains to seed and propagate themselves through host cells could explain the ability of misfolded α-synuclein to act as a prion-like agent, spread from neuron to neuron, and cause disease. Notably, different α-synuclein strains can have different abilities to propagate in cell culture and animal models of Parkinson's disease. Moreover, specific α-synuclein strains may induce similar motor, cognitive, psychiatric, and autonomic signs and symptoms of human Parkinson's disease. The heterogeneity of α-synuclein strains may, therefore, contribute to the variety of clinical presentations of the disease. However, the clinical associations of specific α-synuclein strains are mostly indirect and based on correlational studies. Thus, α-synuclein strains might serve as useful biomarkers of disease prognosis and stratification, although the evidence for their role in Parkinson's disease is still limited. At the same time, there are significant methodological limitations to current approaches used for characterizing α-synuclein strains, and much work remains to be done before α-synuclein strains can be reliably used to predict patient outcomes.

## Introduction and background

α-Synuclein, a neuronal protein consisting of 140 amino acids, is encoded by the SNCA gene, mainly localized at presynaptic terminals. In this manuscript, SNCA stands for the gene, whereas α-synuclein stands for the protein, following the HUGO Gene Nomenclature Committee (HGNC) recommendations. This protein regulates synaptic vesicle trafficking, promotes neurotransmitter release, and participates in SNARE complex formation [1,2]. α-Synuclein could exist in different forms, in particular in soluble monomers and membrane-bound multimers, which allow maintaining synaptic homeostasis [1]. It was reported that α-synuclein misfolding into β-sheet-rich oligomers and fibrils was involved in synucleinopathies [3].

Parkinson’s disease (PD) is the second most common neurodegenerative disorder, which is characterized by the progressive loss of dopaminergic neurons in the substantia nigra pars compacta and the appearance of motor symptoms, such as bradykinesia and resting tremor. Moreover, non-motor symptoms could also develop, including cognitive decline, dysphoria, and autonomic dysfunction [4]. There is strong evidence that α-synuclein aggregation plays a critical role in PD pathogenesis; this process is linked with mitochondrial alterations, lysosomal dysfunction, dysregulated calcium signaling, and neuroinflammation [5,6]. According to the strain hypothesis, α-synuclein could misfold into different pathological conformations, named strains [7]. During the conversion from monomers to fibrils, α-synuclein undergoes multiple steps resulting in different aggregated intermediate structures with distinct toxicities and pathological effects [8]. Several factors contribute to strain formation and could affect SNCA conformation, including gene mutations (A53T, A30P, E46K, H50Q, G51D, A53E), post-translational modifications (PTMs) (Ser129-phosphorylation, truncation, nitration, and ubiquitination) [9]. In addition, environmental influences, such as rotenone, paraquat, mitochondrial toxicity, oxidative stress, and proteasomal or autophagic failure, could also participate in this process [10].

α-Synuclein is a 140-amino-acid protein that was initially identified in the electric organ’s presynaptic terminal and the nucleus [11]. SNCA gene encoding α-synuclein is localized on 4q22.1, whereas protein expression is mainly detected in the central nervous system, particularly in presynaptic terminals [12-14]. Under physiological conditions, α-synuclein is presented as an intrinsically disordered protein (IDP), which is characterized by conformational heterogeneity and the ability to bind different ligands [2,15]. On the one hand, this structural plasticity is essential for the protein’s functional activity, as it promotes the formation of multiple interactions with various binding partners, thereby regulating synaptic vesicle trafficking and neurotransmitter release [16]. On the other hand, α-synuclein’s ability to misfold into distinct pathological conformations makes this protein prone to pathological aggregation [8]. This property is considered one of the key factors in the development of PD and other synucleinopathies.

Physiological functions of α-synuclein are mainly related to the processes of synaptic vesicle trafficking and regulation of dopamine release. It was suggested that SNCA could modulate SNARE complex assembly, thereby controlling neurotransmitter release [1]. Moreover, α-synuclein could bind lipid membranes and adopt an α-helical conformation, which is required for the process of vesicle docking and recycling [17,18]. Interestingly, α-synuclein is not only expressed in the central nervous system but also detectable in peripheral tissues, including erythrocytes, enteric neurons, and olfactory epithelium [19-21]. This fact highlights the possibility of α-synuclein’s involvement in peripheral physiological processes.

α-Synuclein, a neuronal protein consisting of 140 amino acids, is encoded by the SNCA gene, mainly localized at presynaptic terminals. In this manuscript, SNCA stands for the gene, whereas α-synuclein stands for the protein, following the HUGO Gene Nomenclature Committee (HGNC) recommendations. This protein regulates synaptic vesicle trafficking, promotes neurotransmitter release, and participates in SNARE complex formation [1,2]. α-Synuclein could exist in different forms, in particular in soluble monomers and membrane-bound multimers, which allow maintaining synaptic homeostasis [1]. It was reported that α-synuclein misfolding into β-sheet-rich oligomers and fibrils was involved in synucleinopathies [3].

Parkinson’s disease (PD) is the second most common neurodegenerative disorder, which is characterized by the progressive loss of dopaminergic neurons in the substantia nigra pars compacta and the appearance of motor symptoms, such as bradykinesia and resting tremor. Moreover, non-motor symptoms could also develop, including cognitive decline, dysphoria, and autonomic dysfunction [4]. There is strong evidence that α-synuclein aggregation plays a critical role in PD pathogenesis; this process is linked with mitochondrial alterations, lysosomal dysfunction, dysregulated calcium signaling, and neuroinflammation [5,6]. According to the strain hypothesis, α-synuclein could misfold into different pathological conformations, named strains [7]. During the conversion from monomers to fibrils, α-synuclein undergoes multiple steps resulting in different aggregated intermediate structures with distinct toxicities and pathological effects [8]. Several factors contribute to strain formation and could affect SNCA conformation, including gene mutations (A53T, A30P, E46K, H50Q, G51D, A53E), post-translational modifications (PTMs) (Ser129-phosphorylation, truncation, nitration, and ubiquitination) [9]. In addition, environmental influences, such as rotenone, paraquat, mitochondrial toxicity, oxidative stress, and proteasomal or autophagic failure, could also participate in this process [10].

α-Synuclein is a 140-amino-acid protein that was initially identified in the electric organ’s presynaptic terminal and the nucleus [11]. SNCA gene encoding α-synuclein is localized on 4q22.1, whereas protein expression is mainly detected in the central nervous system, particularly in presynaptic terminals [12-14]. Under physiological conditions, α-synuclein is presented as an intrinsically disordered protein (IDP), which is characterized by conformational heterogeneity and the ability to bind different ligands [2,15]. On the one hand, this structural plasticity is essential for the protein’s functional activity, as it promotes the formation of multiple interactions with various binding partners, thereby regulating synaptic vesicle trafficking and neurotransmitter release [16]. On the other hand, α-synuclein’s ability to misfold into distinct pathological conformations makes this protein prone to pathological aggregation [8]. This property is considered one of the key factors in the development of PD and other synucleinopathies.

Physiological functions of α-synuclein are mainly related to the processes of synaptic vesicle trafficking and regulation of dopamine release. It was suggested that SNCA could modulate SNARE complex assembly, thereby controlling neurotransmitter release [1]. Moreover, α-synuclein could bind lipid membranes and adopt an α-helical conformation, which is required for the process of vesicle docking and recycling [17,18]. Interestingly, α-synuclein is not only expressed in the central nervous system but also detectable in peripheral tissues, including erythrocytes, enteric neurons, and olfactory epithelium [19-21]. This fact highlights the possibility of α-synuclein’s involvement in peripheral physiological processes.

## Review

Misfolding and aggregation in disease

Pathological SNCA changes its conformation from a mostly unstructured or α-helical monomer to β-sheet-rich oligomers and fibrils [22,23]. This process of misfolding is a primary event in the pathogenesis of synucleinopathies such as PD, dementia with Lewy bodies (DLB), and multiple system atrophy (MSA) [24,25]. Genetic mutations in the SNCA gene, including A53T, A30P, E46K, H50Q, G51D, or A53E, also promote SNCA aggregation and accelerate disease onset when inherited from a parent or in combination with gene duplication or triplication events [26-30].

Exposure to environmental toxins, including the pesticides rotenone and paraquat, oxidative stress, mitochondrial dysfunction, or deficiencies in the proteasomal or autophagic clearance pathways can also induce aggregation [10,31]. Toxicity caused by misfolded SNCA aggregates involves multiple mechanisms, which comprise the disruption of cellular membranes, mitochondrial depolarization, dysregulation of calcium within the cell, synaptic dysfunction, and the activation of neuroinflammation via microglial-mediated neuroinflammation [32-34]. 

Concept of protein strains

The concept of a protein strain came from prion research [35], when it became apparent that variants exist of the same protein, although they are arranged or structured differently, self-propagated, and have subtle but important distinctions with respect to the resulting disease (i.e., infectivity, transmissibility, and tissue preference for a phenotype) [36]. The original idea that SNCA may be prion-like was based upon the observation that misfolded SNCA aggregates can act as templates for misfolding native SNCA [37,38], effectively seeding the conversion of native SNCA into the same 'seeded' misfolded form, therefore propagating the pathology.

These strains differ in many ways, including fibril morphology, stability, seeding ability, and how they prefer to interact with cell types in the brain. It has been shown that when injected in an animal model, various SNCA fibril conformers can cause different phenotypes from a neuropathological perspective, including some strains that prefer dopaminergic neurons and some strains that prefer oligodendrocytes [9,39].

SNCA strains in synucleinopathies

PD, MSA, and DLB are neurodegenerative diseases characterized by α-synuclein pathology; however, they differ in clinical presentation, natural history, and pathology distribution. This review focuses on PD and MSA, which have been studied most intensely, while data on DLB are limited, although they are briefly mentioned for comparison. Indeed, when comparing strains of α-synuclein fibrils isolated from brain tissue of patients with MSA and PD using state-of-the-art cryo-EM structural analysis, the MSA-related SNCA fibrils were found to have a unique, compact structure at a molecular level that differs from that of PD fibrils [40].

Additionally, using biochemical approaches, such as real-time quaking-induced conversion (RT-QuIC) and protein misfolding cyclic amplification (PMCA), it was shown that seeds of α-synuclein fibrils isolated from patients with MSA caused increased propagation of pathology in oligodendroglial cultures compared with α-synuclein seeds isolated from patients with PD [41,42].

Pathological strain differences of SNCA have significant clinical implications, as these strain weightings may explain the different courses of PD and MSA. In MSA, SNCA strains preferentially target oligodendrocytes, causing widespread deposits of glial cytoplasmic inclusions (GCIs) and a rapid clinical course with early and severe autonomic dysfunction. Strains associated with PD primarily affect nigrostriatal neurons, resulting in a slower course, which is initially motor dominant. These mechanistic differences may help explain the more fulminant phenotype of MSA than the slower clinical course of PD [43-45].

Mechanisms of strain diversity

Strain development of α-synuclein is dictated by both endogenous and exogenous factors. Indeed, some of the described strain variations are likely to be genuine, while others may simply result from differences in preparation conditions, such as the fibril preparation protocol, the nature of the co-factors present, sonication, fibril fragmentation, or differences in assay conditions used during the amplification step. Strain variations could also be caused by PTMs regulating C-terminal truncation, which are involved in the phosphorylation state of serine 129, nitration, and ubiquitination affecting fibril structure and toxicity [46-48], or fibrillogenesis parameters such as lipid membranes, ionic strength, or pH [49].

There is an emerging body of evidence indicating that SNCA does not aggregate in vacuum and is affected by a range of molecular co-factors that provide a dynamic chemical environment for the conformational energetics of SNCA [50]. Even for SNCA strains produced in solitude, the heterogeneity in strains among synucleinopathies and possibly PD (the clinicopathological variations of PD, DLB, or MSA) could also be a product of SNCA interactions with one or more host factors modulating aggregation [51].

Cross-seeding studies have shown that tau fibrils can accelerate the aggregation of SNCA by nucleating the formation of some distinct structure altogether with greater cytotoxicity, contributing to its overall complexity [52]. Some amyloid-β variants have been shown to nucleate SNCA fibril formation, which could contribute to the high comorbidity of both PD and Alzheimer's pathology and lends some explanation regarding the more rapid cognitive decline seen in PD with dementia [53,54]. These protein-protein interactions are indicative that the heterogeneity of SNCA strains may be strongly modulated by the molecular context within a host brain.

Aside from proteins, lipid bilayers and membrane composition are also significant in determining the identity of SNCA strain. The intrinsic affinity of the protein for negatively charged phospholipids indicates that lipid changes (high or low cholesterol, sphingolipid, cardiolipin) can alter fibril morphology and aggregation propensity [55]. Also, experimental studies show that specific lipid environments have a role in accelerating fibrillization, and they are also solidified with structural traits that dictate tropism to neurons or glia [56].

These observations correlate well with clinical evidence that pathology with rich glial content in MSA could be indicative of the lipid-induced selection of oligodendrocytes’ susceptibility; this susceptibility may be connected to the strain-related variation in the pattern of PTMs of SNCA (e.g., phosphorylation, truncation, nitration, and ubiquitination) that can influence the aggregation and co-factor interactions related to it [57]. For example, serine-129 phosphorylation has been shown to cause β-sheet formation in SNCA, and it affects the fibrillation process. Besides, cleavage of the C-terminal part of α-synuclein eliminates the highly charged, solubilizing C-tail of acidic nature, contributing to both charge neutralization and acceleration of fibrillation rates. Terminal truncation of α-synuclein leads to the elimination of the C-terminus, which results in charge neutralization and an increase in the speed of fibrillization. In particular, C-terminal cleavage activates the hydrophobic NAC region (residues 61-95), thereby increasing the rate of aggregation, allowing the creation of strains with improved seeding efficiency and toxicity [58,59]. It may be possible that a significant change in the structure of aggregated α-synuclein caused by PTMs, along with tau, Aβ, and lipid co-factors, influences the variety of strains in the particular case of the disease and explains the differences in clinical presentations.

In conclusion, the discussion of the relationship between SNCA and its co-factors may provide insights into how strain heterogeneity could be manifested at the molecular level. The interactions between SNCA and its co-factors may determine the structure of the amyloid fibrils, that is, the seeding ability for particular cellular pathways, and/or the preference for certain cellular pathways, resulting in different clinical presentations of the disease (e.g., slower progression and predominance of motor signs in PD versus faster progression and autonomic involvement in MSA). One can hypothesize that strain heterogeneity, seeded by co-factors, could be used for the development of diagnostic tests for different clinical forms of synucleinopathies and the treatment of each form on an individual basis. It is noteworthy that the seeding potential of different strains could be used to develop therapeutics against specific protein conformations. Having formed, protein strains retain their “conformational memory” and have the ability to act as seeds in target cells, thus determining the three-dimensional structure of protein aggregates in these cells [60]. At the cellular level, this can lead to different patterns of lesion distribution, manifestations of neuropathology, and, consequently, the variety of clinical pictures of synucleinopathies and their responses to treatment (Figure 1).

**Figure 1:**
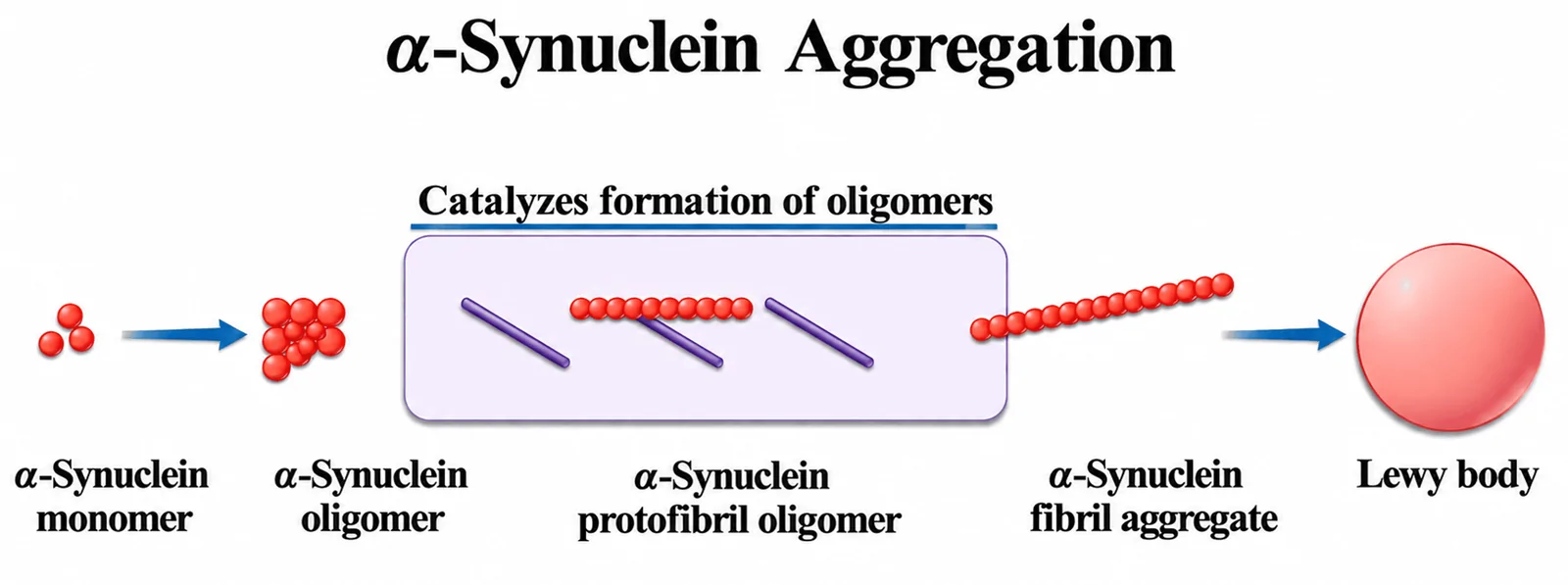
Aggregation pathway of α-synuclein leading to Lewy body formation This figure portrays how α-synuclein progresses from its native monomeric form to oligomers, then proto-fibrillar oligomers, and finally as mature fibrillar aggregates. α-Synuclein oligomers serve as nucleation sites for the (mis)folding of additional proteins and the aggregation of those proteins, catalyzing the development of protofibrils and fibrils. Ultimately, the accumulation and compaction of these fibrillar protein aggregates generate the Lewy Bodies characteristic of Parkinson’s disease and other synucleinopathies. Figure created using Microsoft PowerPoint (Microsoft® Corp., Redmond, WA).

Strain diversity mechanisms

Strain formation is influenced by both internal mechanisms and external mechanisms. The internal mechanisms include PTMs (e.g., S129-phosphorylation, truncation, nitration, ubiquitin modifications), which can alter physical characteristics of fibrils, seeding activity, and toxicity [61,62]. The external mechanisms comprise environmental influences, such as pH, ionic strength, and lipid environment. A previous study [63] demonstrated that during fibrillogenesis, environmental factors could "lock in" certain fibril conformations, resulting in a phenomenon termed structural memory - the tendency of a strain to hold onto a specific “fold” in successive rounds of propagation. The presence of other amyloidogenic proteins, such as tau and amyloid-β, has also been found to influence strain behavior, potentially contributing to the mixed pathologies in some cases of PD [53]. In Table 1, we summarize key studies of SNCA strains in PD in experimental models, methods, strain findings, and clinical conclusions.

**Table 1:** Key studies on SNCA strains in PD and related synucleinopathies Cryo-EM, cryogenic electron microscopy; CSF, cerebrospinal fluid; MSA, multiple system atrophy; PD, Parkinson's disease; PMCA, the protein misfolding cyclic amplification technique; RT-QuIC, real-time quaking-induced conversion assay; SNCA, the gene encoding α-synuclein Cell culture, studies based on the use of cultured cells; fibrillization, the process of changing the structure of α-synuclein into fibrillar aggregates and strain, which refers to a specific type of misfolded α-synuclein having specific pathogenic properties; in vitro, studies conducted outside a living organism; seeding, the process whereby pre-existing α-synuclein aggregates stimulate the aggregation of soluble α-synuclein

Model/Method	Key Findings	Clinical Relevance
Genetic linkage study	Identified A53T mutation in SNCA	Established genetic cause of familial PD
In vitro fibrillization	Demonstrated spontaneous SNCA aggregation	Basis for aggregation assays
Cell culture, seeding	Demonstrated prion-like propagation of SNCA	Introduced strain concept to SNCA
Mouse models	Different SNCA strains produce distinct pathologies	Explained heterogeneity in synucleinopathies
RT-QuIC assay	Discriminated PD vs. MSA SNCA in CSF	Potential diagnostic biomarker
Cryo-EM	Resolved PD vs. MSA fibril structures	Structural basis for strain differences
PMCA assay	MSA seeds amplify more efficiently than PD seeds	Supports clinical progression differences

Review methodology

This study was designed as a retrospective, literature-based analysis that synthesized published research on the SNCA strains in PD and related synucleinopathies. Our process followed established methods for systematically gathering data from neuropathological, biochemical, and structural studies [64], using a focus on studies that utilized strain-discriminating assays. Based on the predefined eligibility criteria, the studies were screened at the title/abstract stage. Exclusion reasons were recorded. The general process of study selection is shown in the PRISMA 2020 flow diagram (Figure 2).

**Figure 2:**
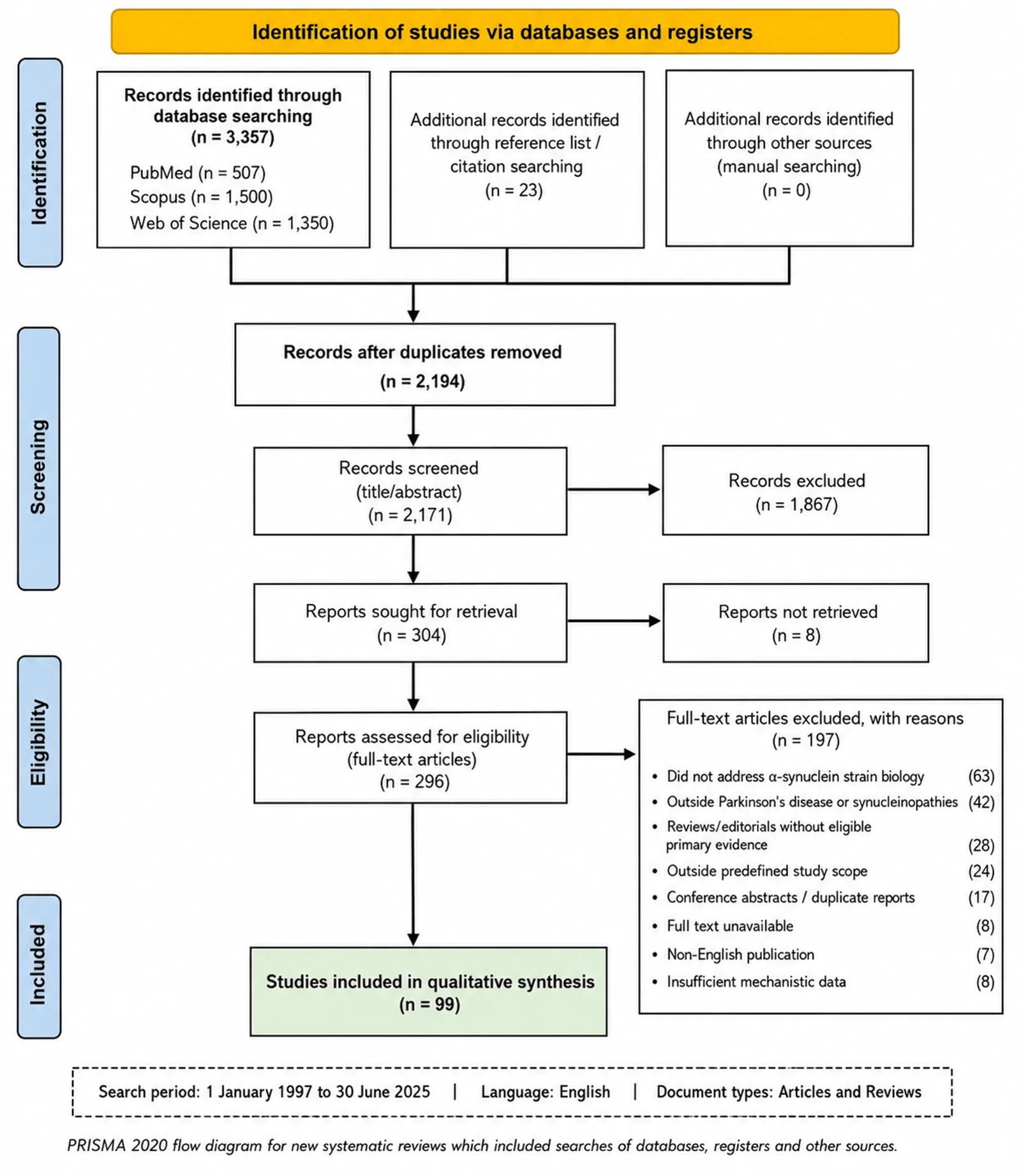
PRISMA 2020 flow diagram illustrating the identification, screening, eligibility assessment, and inclusion of studies in the review of α-synuclein strain biology in Parkinson’s disease and related synucleinopathies. Summary of records identified through PubMed, Scopus, and Web of Science, additional records identified through reference-list/citation searching, removal of duplicates, title/abstract screening, full-text assessment, reasons for exclusion, and the final studies included in the qualitative synthesis. The literature search covered publications from 1 January 1997 to 30 June 2025 and was restricted to English-language articles and reviews.

Literature search strategy

The PubMed, Web of Science, and Scopus databases were searched for studies published from January 1997 to June 2025 (the search was completed on June 30, 2025). The search terms were “SNCA ” or “α-synuclein” and “strain” or “conformer” or “polymorph” and “Parkinson’s disease” or “synucleinopathy” or “ multiple system atrophy” or “Lewy body.” Boolean operators were used to narrow the search, and specific studies were filtered using inclusion criteria such as those published in English and peer-reviewed journals.

Inclusion and exclusion criteria

The research studies included in this analysis were original research articles that were utilized to model human, animal, and/or cell culture. Studies were required to clearly show structural, biochemical, or functional differences between α-synuclein (SNCA) strains. Studies were also required to use advanced detection techniques such as cryogenic electron microscopy (Cryo-EM), RT-QuIC, PMCA, or solid-state nuclear magnetic resonance (NMR). Also included were studies that presented any data comparing strain features of SNCA to clinical/pathological data collected on PD patients.

Exclusion criteria were applied to any study that presented information about expression of SNCA alone with no aggregations or analysis related to strains. Non-peer-reviewed literature was not accepted for this review. Conference abstracts submitted without a full-text publication were also excluded from this review. A final exclusion criterion was applied to any material that does not contain any primary data, including opinion or narrative review articles.

Data extraction and categorization

Two independent reviewers assessed the eligibility criteria and extracted the data that were summarized in the table below according to the following categories: the source and type of SNCA aggregate (human and brain-derived, recombinant fibrils), analytical approach (structural, biochemical, and seeding assay), strain properties (morphology, seeding potency, cell tropism, and stability), and clinical relevance (associated pathology, stage of the disease, symptomatology). The data are presented in Table 2 for comparison. For individual studies, the key methodological information has been included to aid in the replication of the study and the critical assessment of the findings: the population or model system, sample size, stage of the disease, type of α-synuclein species, preparation procedure, assay, the most relevant strain property identified, and limitations of the study, if any.

**Table 2:** Data categorization framework for SNCA strain studies Cryo-EM, cryogenic electron microscopy; MSA, multiple system atrophy; NMR, nuclear magnetic resonance; PD, Parkinson’s disease; PMCA, the protein misfolding cyclic amplification technique; RT-QuIC, real-time quaking-induced conversion assay; SNCA, the gene encoding α-synuclein Clinical correlation, the link between strain features shown by α-synuclein and type of disease state; fibril twist pitch, defining the helical pitch of α-synuclein fibrils; protease resistance, a measure of how resistant α-synuclein aggregates are to proteolysis; thermodynamic stability, comparing the activity of α-synuclein conformations

Parameter	Example Categories
Source of SNCA	PD brain tissue, MSA brain tissue, recombinant protein
Analytical technique	Cryo-EM, solid-state NMR, RT-QuIC, PMCA
Strain-specific features	Fibril twist pitch, thermodynamic stability, protease resistance
Clinical correlation	PD slow progression, PD rapid progression, MSA

Quality assessment

The included studies in the current review were evaluated using an adapted and modified Newcastle-Ottawa Scale (NOS), which integrated the items of the original scale [65]. Specifically, the quality of each study included in the analysis was assessed by using three domains: (1) selection domain (with a score range from 0 to 4) - representing aspects related to the representativeness of the cohort/experimental design under investigation, and the adequacy of the used comparators, validity of the strain description, and subjects’ baseline status; (2) comparability domain (with a score range from 0 to 2) - representing the degree of comparability among the exposed and unexposed groups, depending on the study design and adjustment for potential confounders; (3) outcomes domain (with a score range from 0 to 3) - representing the validity of the strain-specific outcome (follow-up and adequacy of outcomes). The maximum score was 9, while studies with a score ≥6 were considered high-quality studies and given priority in the final synthesis of results.

Data synthesis

As there was considerable diversity among research methods, experimental models, and outcomes, quantitative synthesis through meta-analysis was impossible. For this reason, a narrative synthesis method was used [66]. Important results have been incorporated thematically to answer the main research question: what are the structural and functional differences between different α-synuclein strains, and how do these differences affect the pathogenesis and clinical variability of PD? Although there were conflicting results between studies, they were not neglected or ignored, but rather highlighted and explained using information that takes into account methodological variations, model systems, and experimental settings.

An overview of the overall methodological workflow can be seen in Table 3, which details the sequential framework from the research question to narrative synthesis.

**Table 3:** Methodological framework for the review of α-synuclein strain heterogeneity Cryo-EM, cryogenic electron microscopy; NMR, nuclear magnetic resonance; NOS, Newcastle-Ottawa Scale; PD, Parkinson's disease; PMCA, the protein misfolding cyclic amplification technique; RT-QuIC, real-time quaking-induced conversion assay; SNCA, the gene encoding α-synuclein Cellular tropism, the practice of a particular α-synuclein strain to act in a different manner when it comes to how it interacts with or gets into the cells specifically; seeding efficiency, how well one α-synuclein aggregate helps convert or aggregate dissolving α-synuclein; strain heterogeneity, the phrase used to indicate that there are different variants of α-synuclein aggregates

Step	Methodological Component	Description
1	Research question	How do SNCA strains differ structurally and functionally, influencing PD pathogenesis and clinical variability?
2	Literature search strategy	Databases: PubMed, Scopus, Web of Science. Search terms: “SNCA” OR “α-synuclein” AND “strain”, “conformer”, or “polymorph” AND “Parkinson’s disease”, “synucleinopathy”, “multiple system atrophy”, or “Lewy body”. Filters: English-language, peer-reviewed articles published between 1997 and June 2025.
3	Screening of studies	Inclusion criteria: Original studies (human, animal, or cell culture) investigating structural, biochemical, or functional differences among α-synuclein strains; use of advanced analytical techniques (cryo-EM, RT-QuIC, PMCA, solid-state NMR); and studies with pathological or clinical correlations. Exclusion criteria: Strain analyses not performed, non-peer-reviewed articles, conference abstracts, and studies lacking primary data.
4	Data extraction and categorization	Extracted information included origin and type of α-synuclein aggregate, analytical methods, strain-specific characteristics (morphology, seeding efficiency, cellular tropism, stability), and clinical associations (disease type, progression, symptomatology). Data were categorized according to a predefined framework.
5	Quality assessment	Methodological quality was assessed using a modified NOS. Evaluation criteria included reproducibility, sample size, blinding, and relevance to Parkinson’s disease pathology.
6	Data synthesis approach	Owing to heterogeneity in study designs and outcomes, findings were synthesized using a narrative approach. Key observations were integrated thematically across studies.
7	Outcome	Development of an integrated understanding of α-synuclein strain heterogeneity and its influence on PD pathogenesis, clinical variability, biomarker discovery, and therapeutic development.

Structural diversity of SNCA strains

High-resolution structural research has revealed the existence of non-uniform SNCA fibrils that vary by their protofilament number, helical pitch, and β-sheet properties. For instance, high-resolution cryo-EM analysis of isolated protofilaments obtained from post-mortem PD cerebral tissue demonstrated that they possess a two-protofilament arrangement with salt bridges linking the filaments together [67]. Meanwhile, Schweighauser et al. [40] demonstrated that MSA-derived fibrils had a more compact asymmetric conformation with fewer solvent-exposed residues, making them more resistant to proteolytic digestion. Moreover, Bousset et al. [63] showed that fibrils could be formed by templated seeds that are resistant to multiple rounds of proteolytic digestion in vitro and could be propagated throughout several stages of cell culture. These findings support the templated model of protein misfolding since different conformations of fibrils dictate the misfolding of endogenous α-synuclein monomers by seeded aggregation. This templating process explains the strain-specificity observed during the propagation of pathological features from patient to patient. Indeed, Tuttle et al. [68] applied solid-state NMR to characterize the conformational differences between fibrils with distinct pathological potential at a moderate resolution. The researchers demonstrated that the hydrophobic core and the hydrogen bond propensity of the β-sheets varied significantly between the examined strains. Altogether, the discussed studies highlight the existence of diverse structural conformations of α-synuclein fibrils revealed by cryo-EM. Nevertheless, it is crucial to differentiate structural strains from the biological ones, which can only be characterized by their transmission potential, cellular tropism, and symptomatology in vivo. In other words, structural research contributes to the understanding of fibril conformation on a molecular level, but it might not fully recapitulate the complexity of biological processes that happen inside cells.

Biochemical and seeding properties

Biochemical assays also showed that the SNCA strains exhibited differences in protease digestion profiles, stability to thermal denaturation, and aggregation kinetics. MSA-derived fibrils were consistently more resistant to proteinase K digestion than PD-derived fibrils. This finding is indicative of a more stable fibril core of the MSA-derived strain [69,70]. Thermodynamic stability tests found that fibrils generated from MSA strains retained their fibrillar morphology after heat treatment or exposure to chaotropic agents, while PD strains exhibited partial dissociation after similar treatments [71,72].

In seeding assays, both PMCA and RT-QuIC provided quantitative evidence for strain-specific propagation efficiencies, as MSA-derived seeds rapidly induced fibril formation and had shorter lag phases and higher plateau fluorescence in Thioflavin T (ThT) kinetics, suggesting a higher intrinsic nucleation rate [73,74]. These co-factors have unique biochemical signatures, which may be useful as diagnostic biomarkers and may also help differentiate between different α-synucleinopathies [75].

Proteomic analyses of seeded aggregates have also shown that the fibrils recruit different co-factors, such as lipids and small heat shock proteins, affecting both aggregation kinetics and toxicity [76]. These unique biochemical signatures offer hope as diagnostic biomarkers to differentiate the range of α-synucleinopathies [75].

Cellular tropism and pathogenic spread

Strain diversity extends beyond their biochemical characteristics to cell-type specificity and anatomically diverse spreading of pathology. For instance, in rodent inoculation models, PD-derived fibrils caused intraneuronal inclusions inside the dopaminergic neurons of the substantia nigra pars compacta (in the midbrain) and progressive motor deficits [77,78]. MSA fibrils preferentially targeted oligodendrocytes, causing GCIs and demyelination of white matter tracts [79].

In vitro studies of human induced pluripotent stem cell (iPSC)-derived neurons and glia revealed that the differences in tropism could be related to the interactions between strain-specific fibrils and cell membranes, as well as the entry pathways [80]. For instance, SNCA strains linked with PD were internalized into neurons via clathrin-mediated endocytosis, whereas MSA strains utilized macropinocytosis to invade oligodendrocytes [81]. At the same time, it should be noted that cellular invasion could depend on several factors beyond the strains, including cell types, fibril preparation procedures, fibril size, receptor abundance, experimental conditions, and other host-cellular factors.

In vivo tracking studies have demonstrated that SNCA strains associated with PD generally followed the nigrostriatal propagation pattern, whereas those related to MSA invaded the brain through the corticospinal and cerebellar circuits [82-84]. This difference in propagation was presumably due to the dissimilarities in synaptic connectivity and the ability of specific strains to be transported within axonal tracks, as shown in rodent models and in vitro experiments, although the direct evidence in humans is lacking.

It is also acknowledged that the processes of cellular uptake depend not only on the strain but also on the type of cell, preparation of fibrils, size of the aggregates, the level of expression of receptors, conditions of the experiment, and other factors related to the cell.

The difference in propagation patterns helps to explain the difference in clinical manifestations displayed by PD and MSA, both of which are caused by SNCA aggregates (Table 4).

**Table 4:** Distinct cellular tropism, propagation patterns, and pathological features of α-synuclein strains in Parkinson’s disease and multiple system atrophy GCI, glial cytoplasmic inclusion; MSA, multiple system atrophy; PD, Parkinson's disease; SNCA, the gene encoding α-synuclein Axonal transport, refers to the movement of protein aggregates through axons of nerve cells; cellular tropism, refers to the process of specific α-synuclein strains targeting particular types of cells; cellular uptake, refers to the mechanism through which α-synuclein aggregates enter into the cell; clathrin-mediated endocytosis, refers to the process through which α-synuclein is taken into the cell depending on its receptor; demyelination, refers to the disruption of myelin in neural fibers; Lewy body, refers to the intraneuronal inclusion of aggregated α-synuclein; macropinocytosis, the process by which extracellular substances are absorbed via the use of actin; propagation, refers to the transfer of misfolded α-synuclein aggregated; strain biology, refers to the role played by different conformations of α-synuclein

Feature	PD-Associated SNCA Strains	MSA-Associated SNCA Strains
Primary cellular target	Dopaminergic neurons of the substantia nigra pars compacta	Oligodendrocytes
Pathological inclusions	Intraneuronal α-synuclein inclusions (Lewy body-like pathology)	GCIs
Affected brain region	Substantia nigra pars compacta and nigrostriatal system	White matter tracts, corticospinal and cerebellar pathways
Experimental evidence	Rodent inoculation studies demonstrate neuronal inclusion formation and progressive motor deficits	Rodent inoculation studies show oligodendroglial pathology, demyelination, and white matter degeneration
Cellular tropism	Preferential affinity for neuronal cells	Preferential affinity for oligodendroglial cells
Cellular uptake mechanism	Predominantly clathrin-mediated endocytosis	Predominantly macropinocytosis
Propagation pattern	Nigrostriatal spreading pathway	Corticospinal and cerebellar pathway propagation
Axonal transport characteristics	Compatible with neuronal synaptic connectivity and nigrostriatal transport networks	Compatible with oligodendroglial and cerebellar/corticospinal networks
Clinical consequences	Progressive parkinsonian motor deficits	MSA-associated motor dysfunction, autonomic impairment, and demyelinating pathology
Biological significance	Produces neuronal degeneration characteristic of PD	Produces oligodendroglial degeneration characteristic of MSA
Implication for strain biology	Strain-specific structure determines neuronal targeting and disease phenotype	Distinct conformational strain drives oligodendroglial tropism and MSA-specific pathology

Clinical correlations

Retrospective data analysis revealed that strain type was linked to clinical phenotype and disease progression rates, as well as the pattern of biomarkers in cohorts of patients whose strain typing was successful. In clinical trials of cohorts of tens or hundreds of patients at early, middle, or late stages of PD, the relationship was established between strain type and rates of progression, asymmetry of motor signs at the beginning of the disease, and a delay in the onset of non-motor symptoms [54,85-86]. In conclusion, the strains in MSA were shown to correlate with symptoms of autonomic impairment at the initial stages, as well as with cerebellar ataxia and more rapid decline, resulting in survival below 10 years from diagnosis. Moreover, it was shown that variations in strain-specific differences also correlated with alterations in CSF biomarkers identified using the RT-QuIC assay, with high sensitivity and specificity in differentiating between MSA and PD based on seeding curves [87].

Strain specificity differences are also correlated with variations in CSF markers that can be detected using the RT-QuIC assay, which has a sensitivity and specificity greater than 90% for the differentiation of MSA from PD using seeding curves, which show the kinetics of seeded protein conversions [88]. This may depend on the sample type and assay conditions as well as patient cohorts. In addition, the assay was able to reveal strain-specific patterns even many years before symptom onset in patients diagnosed later with either PD or MSA, indicating the usefulness of such tests in presymptomatic screening.

Histopathological examination confirmed the relationship between the strain and the phenotype: the PD cases usually exhibited SNCA inclusions which were mostly present in the brainstem nuclei and limbic areas, while MSA cases revealed diffuse oligodendroglial inclusions and myelin pathosis throughout the tract [89].

SNCA is not a pathologic entity but a mixture of different conformational strains, each associated with specific structural, biochemical, and pathological features. This hypothesis fits with previously established prion strain biology concepts and is beginning to be accepted as a way to relate the clinical heterogeneity seen among synucleinopathies, including PD, MSA, and DLB. The evidence suggests that the conformational diversity seen in α-synuclein strains is likely to be an integral part of the pathophysiology, driving the differences in symptomatology, progression, and tissue tropism that are seen. This also has crucial implications for the way that disease pathology, progression, and treatment are studied and understood.

Histopathological studies confirm the traditional relation between strain and phenotype. As for PD, SNCA is formed in the critical nuclei of the brainstem and brain limbic structures. Concerning MSA, a great number of inclusions appear in the neuropil along with a moderate loss of myelinated fiber tracts.

Strain-specific pathobiology

Cryo-EM and ssNMR approaches have revealed interesting differences in overall protofilament structure between various SNCA strains, which likely contribute to the differences seen in disease pathology [90-92]. In particular, PD fibrils tend to have a two-protofilament structure with exposed acidic residues, which may drive their interaction with membranes, whereas MSA fibrils are more compact and have a hydrophobic core, enabling more efficient packing and resistance to degradation [40,93]. In turn, the difference in structure leads to differences in biophysics, with MSA fibrils being more thermodynamically stable, having shorter lag phases during seeded aggregation, and inducing more severe pathology in animal models. By comparison, although they appear less aggressive, the PD fibrils selectively target the metabolically stressed, highly arborized dopaminergic neurons of the substantia nigra pars compacta with high levels of basal oxidative stress, causing a slower, more focal disease progression in this area and related structures. It is notable that such variations in pathology seem to follow from subtle differences in templating, which was demonstrated in vitro for the differences in the seeding potential of various α-synuclein strains [63]. This suggests that the observed structural differences may have physiological relevance, potentially driving the variation in pathophysiology through strain-specific interactions. However, it should be noted that the variation in pathology likely goes beyond simply the templated misfolding of α-synuclein, as differences in host-cellular factors, such as membrane composition, would also affect propagation, and various cellular pathways, including the autophagy-lysosome and ubiquitin-proteasome systems, could affect the toxicity of the protein aggregate.

Cell-type specificity and neuroanatomical tropism of SNCA strains

One of the crucial discoveries in research into synucleinopathies is the fact that various forms of the alpha-synuclein protein exhibit a certain preferential binding to a specific cell type. In the example of the disease known as Parkinson’s, pathogenic forms of the SNCA gene are likely to invade neurons, in particular, neurons in the nigrostriatal pathway, which is responsible for movement disorders. As a consequence of this, various "classical" pathogenic structures form in neurons, such as Lewy bodies and Lewy neurites [94]. Conversely, in multiple system atrophy, the pathogenic proteins from the SNCA gene act selectively on oligodendrocytes, resulting in the destruction of myelin surrounding the axons and the rapid development of disease marked by rapid formation of GCIs and rapid breakdown of myelin [39].

The characteristics of infected cells are based on many biophysical, biochemical, and biological aspects. It seems that the effect of strain is due to the structural differences of a strain that trigger differences in surface charge and hydrophobic properties of different strains compared to the protein naturally present in the human body. These differences lead to recognition and uptake of α-synuclein strains by specific cells. According to recent studies, cell surface receptors, including LAG3, LRP1, and heparan sulfate proteoglycans (HSPGs), facilitate the selective binding of α-synuclein strains prior to their entry into cells, which could explain the molecular mechanisms of differential cell tropism. In addition, the pathways by which α-synuclein enters cells vary among the strains; namely, PD strains enter the cells by means of the clathrin-dependent mechanism, while MSA strains use macropinocytosis, which is a nonselective aggressive endocytosis method utilized by oligodendrocytes [81].

Different forms of intracellular trafficking and axonal transport of SNCA strains help in determining the ways in which these forms are spread. The strains of Parkinson's disease do not deviate from the original routes of spread of nigrostriatal and limbic pathways in terms of motor and non-motor symptoms. MSA strains spread in tangential directions through other pathways corresponding with cerebellar ataxia, autonomic physiological changes, and fasting effects in the clinical picture [83]. These variances from each other are an example of the main idea that the structure of a strain determines the types of cells which can be attacked, as well as the course that its infection follows through the body. Hence, knowledge about the peculiarities of the spread of various strains of synucleinopathies should help in understanding their pathology shows different clinical symptoms, and will demonstrate the significance of discovering the solution to the problem of using such peculiarities for making diagnosis or implementing treatment methods.

Diagnostic potential and clinical translation

From the translation perspective, the method of SNCA strain profiling has diagnostic potential. It can be used to differentiate between synucleinopathies on the conformational basis. It is critical for implementing the appropriate therapeutic strategy for each patient. In other words, it enables the personalized medicine approach, which is particularly needed for PD due to the heterogeneity of the disease. Moreover, biochemical assays allow for detecting disease biomarkers at early stages and even before symptom onset. The application of biochemical assays to detect pathological α-synuclein aggregates using RT-QuIC or PMCA revealed that they can discriminate between PD, MSA, and other synucleinopathies with an overall sensitivity and specificity that usually exceed 90% [41,88]. However, it should be noted that these data vary depending on the CSF or other sample types and study designs. Indeed, strain-specific profiles were found in the CSF samples of some patients prior to the appearance of their clinical symptoms. Before implementation in the clinic, these assays need to be validated in large-scale studies with diverse populations, and factors influencing false-positive results, such as age and other biomarkers, should be taken into account. This represents a promising emerging application, as it may allow for screening at-risk populations within the so-called neuroprotective window, pending further validation. Moreover, such assays could be critical for designing clinical trials since they enable the appropriate stratification of patients, which will ultimately minimize the time needed for each trial and reduce costs. Individual responses to therapies could also be defined based on the strain-specific conversion patterns.

Therapeutic implications

The strain paradigm is generally challenging for therapeutic strategies, which are largely ineffective against different varieties of synucleinopathies. Even such an approach as immunotherapy, including monoclonal antibodies [95], may be limited by the ability to recognize epitopes masked by specific conformation of aggregated proteins in different species. Small molecule aggregation inhibitors and peptide-based drugs are also considered as a choice for tackling the diversity of strains, targeting the key structures for every disease.

With regard to MSA, the current understanding of the disease suggests that there are oligodendrocytes connection specificities. Therapeutic intervention may be limited by the condition of oligodendrocytes, because the treatment may lead to myelin destruction to induce proteostasis in oligodendrocytes or glial cells, or to inhibit the strain by glial uptake, while for Parkinson’s disease, the strategy could be aimed at neurons or maintaining the dopaminergic pathway.

Current gaps and future research priorities

The still-emerging field of SNCA strain biology poses many questions in need of better characterization. First, what is the nature of the diversity? Genetic determinants (e.g., SNCA mutation status), PTMs (phosphorylation, truncation), environmental co-factors, or “stochastic” events governing misfolding may underlie the observed heterogeneity. Ongoing longitudinal studies in patients will undoubtedly challenge some of the assumptions about strain conversion during the course of disease and define whether specific strains drive phenotypical change, similar to what has been observed in prion disease [96]. Indeed, while multiple robust experimental systems have been developed and utilized to interrogate this question (injections of pre-formed fibrils into rodents, intra-cerebral injections of MSA patient brain extracts into thy1-hSNCA transgenic mice such as M83, or human iPSC-derived neuronal-glial cultures), future work is needed to adapt these approaches to MSA and determine whether, when, and how strain conversion occurs. Likewise, studies utilizing more human-relevant systems such as 3D organoid culture containing multiple cell types, including glia and immune cells, would be of great interest to the field. Lastly, development of accessible diagnostic assays to define strain profile is a critical unmet need. While interesting recent work has utilized minimally invasive nasal mucosa or skin biopsies and RT-QUIC to type prion strains, similar approaches represent attractive options to stratify MSA cases by strain, though they require additional validation in natural disease.

## Conclusions

This retrospective analysis shows that SNCA strains represent an important factor contributing to the variation in clinical presentation, pathology, and biochemistry of synucleinopathies. SNCA aggregates are not simply one pathological process, but groups of conformationally reduced SNCA with heritable structural and functional properties. Polymorphic SNCA strains differ in proteostability, seeding kinetics, cellular tropism, and neuroanatomical propagation, thereby resulting in clinical phenotypes related to human disease and loss of dopaminergic neurons in PD and oligodendroglial inclusions in MSA.

The translational implications of these findings are profound. Strain-specific seeding assays, such as RT-QuIC and PMCA, are ultra-sensitive diagnostics and can distinguish between PD, MSA, and the other related disorders, possibly before clinical stages. These modalities will allow us to better identify risk, more efficiently pre-screen primary candidates for possible efficacy/effectiveness, and target treatments with variable precision. However, if we accept the strain-morphological underpinnings of diversity, this will also complicate drug development; antibodies, aggregation inhibitors, and neuroprotective modalities are not simply conformer-specific; they also have demonstrated strain conformer scarcity. Future organized and directed attempts, using high-resolution structural biology and amplification-type diagnostics to new rubrics of disease, will ultimately be needed to identify the origin, change, and clinical and prognostic relevance of SNCA strains.
